# Changes in corneal symmetry and tear film stability after laser asymmetric keratectomy in patients with keratoconus suspect: A retrospective study

**DOI:** 10.1097/MD.0000000000045488

**Published:** 2025-10-31

**Authors:** Ji Sang Min, Byung Moo Min

**Affiliations:** a Department of Ophthalmology, The Institute of Vision Research, Yonsei University College of Medicine, Seoul, Republic of Korea; b Woori Eye Clinic, Affiliated Clinical Professor to Department of Ophthalmology, Yonsei University College of Medicine, Daejon, Republic of Korea.

**Keywords:** corneal remodeling, corneal symmetry, keratoconus suspect, laser asymmetric keratectomy, postoperative corneal ectasia, visual outcomes

## Abstract

Keratoconus suspect (KCS) has been a contraindication of laser refractive surgery (LRS) due to postoperative corneal ectasia, but is well indicated with LRS combined with corneal remodeling using laser asymmetric keratectomy (LAK). This study compared the 2-year changes in corneal symmetry and tear film stability after laser epithelial keratomileusis-LAK (L-LAK) for new corneal remodeling in myopic patients with KCS. This retrospective case-control study included 31 eyes of 21 patients with KCS who had undergone L-LAK for corneal remodeling. L-LAK procedures included correction of original refractive errors (original ablation), ablation of the thick peripheral cornea to improve the corneal shape, and simultaneous central ablation to correct myopic shift (crescentic customized ablation). Preoperative and 2-year postoperative corneal symmetry were both evaluated using the total corneal central-thickness deviations in 4 directions on an Orbscan map (SUM) (in µm). Two years postoperatively, the average spherical equivalent was decreased from −2.60 ± 1.02 preoperatively to −0.41 ± 0.33 D postoperatively (*P* = .001). The uncorrected distance visual acuity (UDVA) (LogMAR) was increased from 0.82 ± 0.30 to 0.04 ± 0.18 (*P* = .001), and 95% of the participants experienced an UDVA of 20/20 or better. The SUM (µm) decreased from 140.86 ± 48.20 preoperatively to 64.20 ± 15.20 postoperatively (*P* = .001). The Kmean, Kmax, and central pachymetry also decreased (*P* = .001). The tear breakup time (TBUT) (seconds) increased markedly from 7.51 ± 2.96 preoperatively to 19.40 ± 3.65 at 2-year postoperatively (*P* = .001). There were no postoperative adverse events. Corneal remodeling using L-LAK is a safe technique that facilitates corneal symmetry, increases TBUT, and yields good postoperative visual outcomes without postoperative corneal ectasia.

## 1. Introduction

Keratoconus suspect (KCS) is one of the 3 types of the earliest-stage keratoconus (keratoconus stage 1). Despite the lack of a standard definition, several reports have indicated the important diagnostic features (Kmax > +47.0 D), including slight bowing on topography and asymmetric corneal thickness.^[1–3]^ True KCS is an absolute contraindication to laser refractive surgery (LRS) because of the possibility of postoperative corneal ectasia based on thinner corneal steepening and corneal biomechanical interactions.^[2]^ LRS involves symmetric corneal ablation, resulting in thinner corneal steepening on the thinner portion of the asymmetric corneal thickness; on the other hand, laser asymmetric keratectomy (LAK) can selectively ablate thicker peripheral corneas and can shape asymmetric corneas to improve their shape, preventing postoperative corneal ectasia in KCS.

Corneal asymmetry is a major cause of complications of LRS.^[3–12]^ As established in our previous studies,^[10–15]^ the total corneal central-thickness deviations in 4 directions on an Orbscan map (SUM), and the distance between the maximum posterior elevation (best-fit sphere) and the visual axis (DISTANCE) are important evaluation indices for corneal asymmetry. In patients with preoperative corneal asymmetry (SUM ≥ 80 µm), corneal remodeling with LAK to improve the corneal shape constitutes a novel approach to avoiding the conventional adverse effects of LRS.^[10–15]^ Recently, LAK, linked with Vision Up software (Well C, FDA-approved in the Republic of Korea), has been introduced as a method for the biomechanical customization of corneal remodeling.^[10–15]^ By selectively ablating thicker peripheral corneas, this novel approach can shape asymmetric corneas to improve their shape and is indicated to prevent LRS complications.^[10–12]^ This new method enables the re-treatment of patients with post-LRS myopic changes,^[13,14]^ allows for the management of postoperative LRS or adverse effects of ocular surgery,^[14]^ and constitutes a new and feasible treatment method for KCS.^[15]^ Furthermore, crescentic keratectomy has yielded good surgical outcomes when used as a customized corneal remodeling technique in KCS and keratoconus.^[16,17]^ A custom method was also attempted based on an intracorneal ring with custom-shaped CAIRS of multiple type segments in keratoconus.^[18]^ LRS-LAK corrected the refractive power, reduced SUM, and yielded good surgical outcomes without postoperative adverse effects in patients with KCS.^[15,16]^

In this study, we evaluated the change in corneal symmetry after laser epithelial keratomileusis-LAK (L-LAK), a new corneal remodeling technique for patients with myopia and KCS.

## 2. Materials and methods

### 2.1. Patients

This study included 36 eyes of 21 patients who were diagnosed with KCS and treated at the Woori Eye Clinic (Daejon, Republic of Korea) between January and December 2020. This retrospective study was approved by the Korean National Institute for Bioethics Policy (Approval No. P01-202011-21-024). This study adhered to the tenets of the Declaration of Helsinki. All patients provided written informed consent for their medical information to be included in this study.

The inclusion criteria were a diagnosis of KCS,^[1–4]^ manifesting both as abnormal localized corneal steepening >47.0 D on the thinner corneal portion and peripheral corneal thickness asymmetry (SUM ≥ 80 µm), with 20/20 in corrected vision, followed by treatment with L-LAK between January and December 2020 at the Woori Eye Clinic (Daejon, Republic of Korea), with at least a 2-year follow-up duration. The exclusion criteria were previous eye operations or problems, corneal scarring, trauma, pregnancy or lactation, glaucoma, non-corneal causes of ocular astigmatism, and a follow-up duration of <2 years.

### 2.2. Surgical technique for L-LAK

All patients with SUM ≥ 80 µm and focal steepening keratometric curvature of >47.0 D on the thinner corneal portion underwent L-LAK using the same method, the same excimer laser machine (Kera Harvest Inc., Taiwan), and the same surgeon (Byung Moo Min).^[10–17]^ The refractive errors were corrected (optic zone: 6.5 mm; transitional zone: 2 mm) after the removal of a 10.0 to 10.5 mm diameter portion of the epithelium.

**Figure 1.**
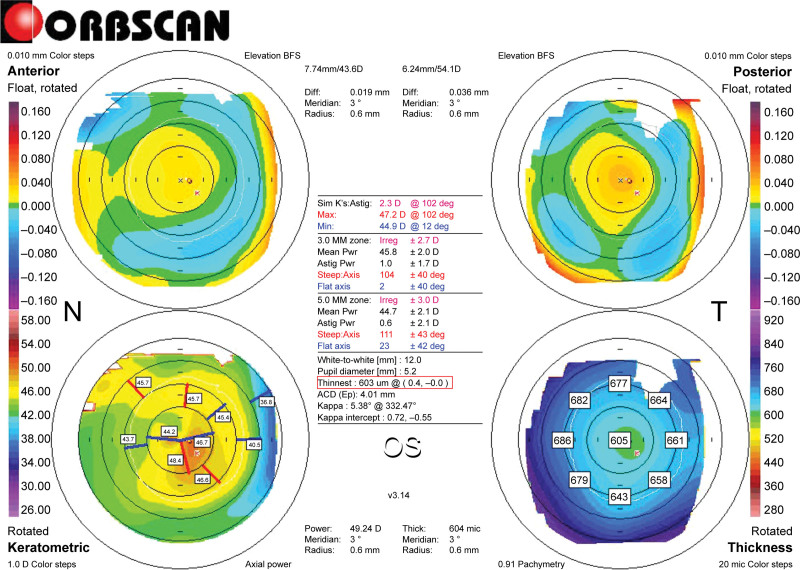
Pre-crescentic L-LAK Orbscan map of Case 1. Right-bottom: A pachymetric map presents an example of the measured differences in thickness between symmetrically opposed points (0–180°, 45–225°, 90–270°, and 135–315°): 0–180°: 25 µm; 45–225°: 15 µm; 90–270°: 34 µm; 135–315°: 24 µm; and SUM: 98 µm. Right-top map: Measurement of the distance between the maximum posterior elevation (best-fit-sphere; BFS) and the visual axis. Corneal apex: temporally deviated (right upper red circle). The thinnest point (X, Y) is indicated by the lower red square, and the posterior high point on thicker cornea and the front elevation on the thinner cornea are presented. DISTANCE: 0.4 mm, Kmax: +48.4 D. L-LAK = laser epithelial keratomileusis-LAK.

**Figure 2.**
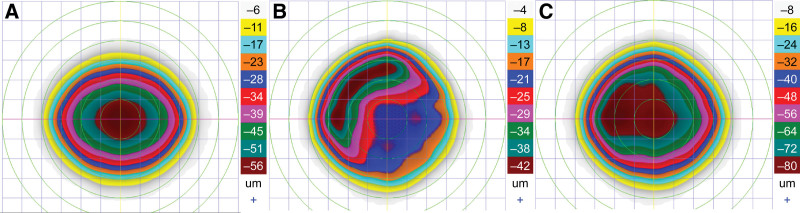
L-LAK ablation planning of Case 1. (A) Original ablation plan of refractive errors (−2.50 = −1.25 180°). (B) Crescentic customized ablation of both the thicker peripheral cornea evaluated by Vision Up software (Well C, Republic of Korea) on Orbscan II (Bausch & Lomb, Bridgewater) corneal maps and central cornea, for the LAK-induced myopic change. (C) Crescentic L-LAK ablation plan (total ablation = original ablation + customized ablation + ablation of LAK-induced myopic shift). LAK = laser asymmetric keratectomy, L-LAK = laser epithelial keratomileusis-LAK.

**Figure 3.**
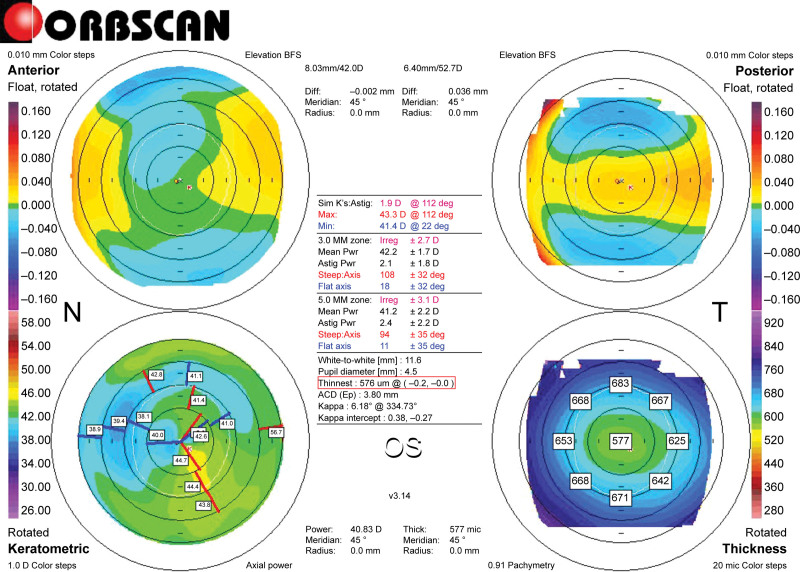
Post-L-LAK Orbscan map of Case 1. Right-bottom: Pachymetric map: an example of the differences in thickness between symmetrically opposed points is shown (0–180°, 45–225°, 90–270°, and 135–315°): 0–180°: 28 µm; 45–225°: 1 µm; 90–270°: 12 µm; 135–315°: 26 µm; SUM: 67 µm. Right-top map: indicates the measured distance between the maximum posterior elevation (best-fit-sphere; BFS) and the visual axis. Corneal apex: temporally deviated (right upper red circle). The thinnest point (X, Y) is indicated by the lower red square. Distance: 0.2 mm. Kmax: +44.7 D. L-LAK = laser epithelial keratomileusis-LAK.

To perform L-LAK,^[10–15]^ Vision Up software (Well C) was interfaced with the laser machine and used to predict the difference between the thicker and thinner corneal thicknesses, while calculating the ablation amounts of the thicker cornea, direction of the axis, and extent of induced myopia that would result from the ablation of the thicker cornea (Figs. 1 and 2). Figure 2 illustrates how L-LAK was implemented, indicating the original ablation (Fig. 2A) for correcting the refractive errors, crescentic customized ablation (Fig. 2B), and total ablation (both original and crescentic customized ablation; Fig. 2C). This process made the cornea symmetric, corrected the original refractive errors and myopic shift due to crescentic LAK ablation, and allowed for selective ablation of the thicker corneal area. Therefore, the cornea was reshaped to achieve symmetry with an ideal refractive power (Fig. 3 and Fig. S1, Supplemental Digital Content, https://links.lww.com/MD/Q484).^[10–15]^

### 2.3. Pre- and postoperative assessment

Before surgery, all patients underwent comprehensive eye examinations, and topographic images were acquired using Orbscan II (Bausch & Lomb, Bridgewater) corneal maps (Fig. 1).

Intraoperatively, we assessed the optic zones, ablation depths, myopic changes due to ablation of the thicker peripheral cornea, and residual stromal beds.

A retrospective analysis of the preoperative and 2-year postoperative follow-up examination data was performed, assessing the following variables that included spherical equivalent (SE), sphere, cylinder, uncorrected distance visual acuity (UDVA), intraocular pressure (IOP), pupil diameter, keratometry (including Kmean, Kmax, and central pachymetry on the Orbscan map), corneal symmetry (SUM, DISTANCE, and kappa angle), and tear film (tear breakup time [TBUT] and tear osmolarity) (Figs. 1 and 3).^[10–15]^

Pre- and postoperatively an auto refractometer/keratometer (TOPCON, Tokyo, Japan) was used to measure the refractive errors, which were then converted into SE. A 6-m visual acuity chart was used to measure UDVA, which was changed to the logarithm of the minimum angle of resolution (LogMAR) of visual acuity. The TBUT (second) and tear osmolarity were measured using Keratograph 4 (Oculus, Wetzlar, Germany). KCS was diagnosed using corneal topography findings, including a Kmax > 47.0 D or an asymmetric bowtie pattern, a normal-appearing cornea on a slit-lamp biomicroscope, and at least one of the following signs that included SUM ≥ 80 µm, oblique astigmatism >1.50 D, thin central cornea <500 µm, or clinical keratoconus in the fellow eye.^[1,3,4]^

Subsequently, a T-lens was fitted. Topical antibiotics (Vigamox; Alcon, Fort Worth) were instilled every 6 hours for 1 week, and steroid eye solution (Ocumetholone; Samil Pharmaceutical, Seoul, Republic of Korea) was administered every 6 hours for 6 weeks.

## 3. Statistical analysis

Paired *t*-tests were performed for normally distributed variables, and the Wilcoxon signed-rank test was used for non-normally distributed variables. A *P*-value of less than .05 was considered statistically significant. Data analysis was performed using SPSS version 25 (IBM Corp., Armonk).

## 4. Results

In total, 36 eyes of 21 myopic patients (mean age: 35.0 ± 12.4 years, range: 23–47 years) underwent L-LAK for KCS manifesting with a SUM ≥ 80 µm on Orbscan maps and focal steepening of the keratometric curvature to >47.0 D on the thinner corneal portion. The study included 9 male and 12 female patients. Although 15 patients received treatment for both eyes and inter-eye bias within 1.5 D of SD, 6 patients received treatment for only one eye. Intraoperatively, the ablation depths (µm) of the center and periphery were 60.23 ± 21.22 (range: 41–81) and 64.02 ± 16.21 (range: 48–80), respectively, for L-LAK. Furthermore, the change in myopia (D) after LAK was −1.82 ± 0.48 (range: −1.15 to −2.25), and the residual stromal depth (µm) was 440.36 ± 26.20 (range: 412–466) at L-LAK (Table 1).

**Table 1:** Demographic characteristics of the participants.

Item	n = 21 patients (36 eyes)
Age (yr), mean ± SD	35.0 ± 12.4
Male: Female	9:12
Operation of 2 or one eye (s)	15:6
Intraoperative findings, mean ± SD	
Optic zone (mm)	6.40 ± 0.09
Ablation depth (µm)	
Central	60.23 ± 21.22
Peripheral	64.02 ± 16.21
Myopic shift (D) due to LAK	−1.82 ± 0.48
Residual stromal depth (µm)	440.36 ± 26.20

LAK = laser asymmetric keratectomy.

### 4.1. Refraction testing results, vision acuity, and keratometry

After 2 years, the SE (D) decreased from −2.60 ± 1.02 (range: −2.5 to −3.75) preoperatively to −0.41 ± 0.33 (range: 0–0.75) postoperatively (*P* = .001), the Sphere (D) decreased from −2.14 ± 4.32 (range: −1.25 to −6.75) preoperatively to −0.20 ± 0.40 (range: 0–0.75) postoperatively (*P* = .001), the cylinder (D) decreased from −1.02 ± 2.20 (range: 0.00 to −3.25) preoperatively to −0.41 ± 0.40 (range: 0–0.75) postoperatively (*P* = .003), and the UDVA (LogMAR) improved from 0.82 ± 0.30 (range: 0.50–1.20) preoperatively to 0.04 ± 0.18 (range: 0–0.20) postoperatively (*P* = .001). Moreover, 95% of the cohort presented with UDVA of 20/20 or better. The L-LAK exhibited good visual acuity outcomes, and there was no postoperative cylindrical axial change. The keratometric (Kmean, Kmax) values decreased from +45.20 ± 0.93 and +48.20 ± 0.80 to +42.65 ± 1.82 and +44.45 ± 1.36, respectively (*P*s = .001; Table 2).

**Table 2:** Comparison of the preoperative and 2-yr postoperative findings.

Findings	Preoperative	2-yr postoperative	*P*-value
Refraction			
SE (D)	−2.60 ± 1.02	−0.41 ± 0.33	.001
Sphere (D)	−2.14 ± 4.32	−0.20 ± 0.40	.001
Cylinder (D)	−1.02 ± 2.20	−0.41 ± 0.40	.003
UDVA (LogMAR)	0.82 ± 0.30	0.04 ± 0.18	.001
IOP (mm Hg)	15.65 ± 2.34	15.74 ± 1.30	.059
Pupil size (mm)	4.09 ± 0.71	4.11 ± 0.60	.060
Keratometry			
Kmean	45.20 ± 0.93	42.65 ± 1.82	.001
Kmax	48.20 ± 0.80	44.45 ± 1.36	.001
Pachymetry			
CP (µm)	566.30 ± 31.32	508.67 ± 46.90	.001
Corneal symmetry			
SUM (µm)	140.86 ± 48.20	64.20 ± 15.20	.001
DISTANCE (mm)	1.10 ± 1.13	0.45 ± 0.40	.001
Kappa angle (degree)	4.54 ± 1.20	2.40 ± 1.72	.002
Tear film			
TBUT (s)	7.51 ± 2.96	19.40 ± 3.65	.001
Tear osmolarity (Osm/L)	0.54 ± 0.14	0.22 ± 0.12	.001

CP = central pachymetry, D = diopters, DISTANCE = distance between the maximum posterior elevation (best-fit-sphere [BFS]) and the visual axis, IOP = intraocular pressure, LogMAR = logarithm of the minimum angle of resolution, SE = spherical equivalent, SUM = sum of deviations in corneal thickness in 4 directions on Orbscan map, TBUT = tear break-out time, UDVA = uncorrected distance visual acuity.

### 4.2. Topographic results

The results indicated a significant reduction in central pachymetry (µm), from 566.30 ± 31.32 (range: 530–597) preoperatively to 508.67 ± 46.90 (range: 462–554) postoperatively (*P* = .001). The mean (Kmean) and maximum (Kmax) curvatures reduced significantly from 45.20 ± 0.93 D and 48.20 ± 0.80 D preoperatively to 42.65 ± 1.82 D and 44.45 ± 1.36 D postoperatively, respectively (*P*s = .001; Table 2). Regarding corneal symmetry, the SUM, DISTANCE, and Kappa angle decreased markedly from 140.86 ± 48.20 µm, 1.10 ± 1.13 mm, and 4.54 ± 1.20 preoperatively to 64.20 ± 15.20 µm, 0.45 ± 0.40 mm, and 2.40 ± 1.72 postoperatively, respectively (*P*s < .05). Of the 36 eyes, all (100%) exhibited symmetry (SUM < 80 µm) on Orbscan topographic analysis.

### 4.3. Tear film results

The TBUT (seconds, on average) increased notably from 7.51 ± 2.96 preoperatively to 19.40 ± 3.65 postoperatively (*P* = .001), whereas the tear osmolarity (Osm/L) decreased from 0.54 ± 0.14 preoperatively to 0.22 ± 0.12 postoperatively (Table 2).

### 4.4. IOP, pupil size, and postoperative complications

The IOP and pupil size of the patients were similar in the preoperative and postoperative assessments (*P*s = .059, .060, respectively; Table 2). There were no postoperative complications, such as myopic changes, severe dry eye, halos, or postoperative corneal ectasia.

## 5. Discussion

In patients with KCS, LRS is completely contraindicated because of the risk of postoperative corneal ectasia.^[2]^ Abdollahian et al reported that 6 of 211 eyes (2.84%) in the photorefractive keratectomy group and 5 of 96 eyes (5.20%) in the laser in situ keratomileusis (LASIK) group developed postoperative corneal ectasia.^[17]^ In their study, patients with KCS received only symmetric refractive ablation such as photorefractive keratectomy or LASIK. Cases were reviewed for irregular corneal topographic patterns but not for asymmetric peripheral corneal thickness, which is a major cause of adverse LRS effects.^[3–12]^ SUM and DISTANCE are important evaluation indices of corneal asymmetry.^[10–15]^ In our study, both refractive errors and corneal asymmetry were corrected simultaneously with L-LAK and Vision Up software. Therefore, SE and UDVA exhibited excellent results, and postoperative corneal ectasia was not detected in any of the participants. Postoperative SUM and DISTANCE decreased markedly, resulting in improved corneal symmetry without postoperative corneal ectasia. Preoperatively, uneven corneas with Kmax > 47.0 D at the thinner corneal portion (Kmax = 48.20 ± 0.80 D) achieved even corneal thickness (Kmax = 44.45 ± 1.36 D) postoperatively. If patients exhibit a preoperative SUM ≥ 80 µm, the IOP could cause protrusion of the thinner corneal region, leading to the postoperative adverse effects of LRS.^[6–15]^ Moreover, in Min and Min‘s studies, LASIK with LAK reduced total corneal thickness deviation, improved corneal symmetry, and significantly reduced blurring scores compared with LASIK without LAK in patients with SUM ≥ 80 µm.^[11,12]^ Corneal curvature changes are caused by differences in the distribution of corneal thickness.^[2,5–9]^ SUM and DISTANCE decreased markedly from 140.86 ± 48.20 µm and 1.10 ± 1.13 mm preoperatively to 64.20 ± 15.20 µm and 0.45 ± 0.40 mm postoperatively, respectively. This suggests that L-LAK decreased these parameters. SUM and DISTANCE, which are evaluation indices, were easily calculated using the Orbscan map.^[10–15]^

We employed Orbscan maps for corneal topography visualization rather than the current Sheimpflug techniques. DISTANCE and SUM can only be calculated on Orbscan maps, as they feature a central visual axis and, thus, are very useful for measuring symmetry. However, to avoid false positives and unnecessary treatment, epithelial maps were not performed in any case.

The TBUT increased to >15 s postoperatively, indicating that the postoperative evenness of the cornea maintained the tear layer better than the preoperative uneven cornea.^[19–21]^

We can calculate the differences in corneal thickness and the extent of myopia-related changes induced by customized ablation using this software, facilitating the conversion of an asymmetric cornea to a symmetric cornea (Fig. 2A, original ablation; Fig. 2B, crescentic customized ablation). The result was a symmetric cornea without refractive errors (Figs. 2C and 3 and Fig. S1, Supplemental Digital Content, https://links.lww.com/MD/Q484).^[10–15]^ L-LAK decreased SUM and DISTANCE and resolved the disadvantages of LRS. Therefore, L-LAK can improve visual outcomes in patients with myopia characterized by a SUM ≥ 80 µm on Orbscan maps.^[8–15]^ Nonetheless, we recommend conventional LRS for relatively symmetric corneas (SUM < 80 µm preoperatively).

This study possesses some limitations. We included only a relatively small number of patients with KCS (more than 20 patients) in this comparative study. Therefore, future studies should include longer follow-up evaluations over 5 years to assess both pre- and postoperative changes in biomechanical properties.

In conclusion, L-LAK, a novel customized corneal remodeling technique, facilitates the restructuring of the cornea for symmetry, with excellent visual results in patients with KCS.

## Acknowledgments

We would like to thank Editage (www.editage.co.kr) for statistical analysis and English language editing. We would also like to thank Hye Won Jung, Rag Seon Han, Eun Mi Jang, Ji Yeon Choi, Mi Kyung Kim, Ji Suk Kwon, and Sun Hee Lee at the Woori Eye Clinic for their assistance in ocular examinations.

## Author contributions

**Conceptualization:** Byung Moo Min.

**Data curation:** Byung Moo Min.

**Formal analysis:** Ji Sang Min.

**Investigation:** Byung Moo Min.

**Methodology:** Byung Moo Min.

**Project administration:** Byung Moo Min.

**Resources:** Byung Moo Min.

**Software:** Byung Moo Min.

**Supervision:** Byung Moo Min.

**Validation:** Ji Sang Min, Byung Moo Min.

**Visualization:** Byung Moo Min.

**Writing – original draft:** Ji Sang Min, Byung Moo Min.

## Supplementary Material


