# Supplementary material for: Changes in corneal symmetry and tear film stability after laser asymmetric keratectomy in patients with keratoconus suspect: A retrospective study
Source: Medicine (Baltimore). 2025 Oct 31;104(44):e45488. doi: 10.1097/MD.0000000000045488 (PMC12582715; doi:10.1097/MD.0000000000045488)
Supplement: Supplementary file 1 [file medi-104-e45488-s001.docx]

**Supplemental Digital Content**

**
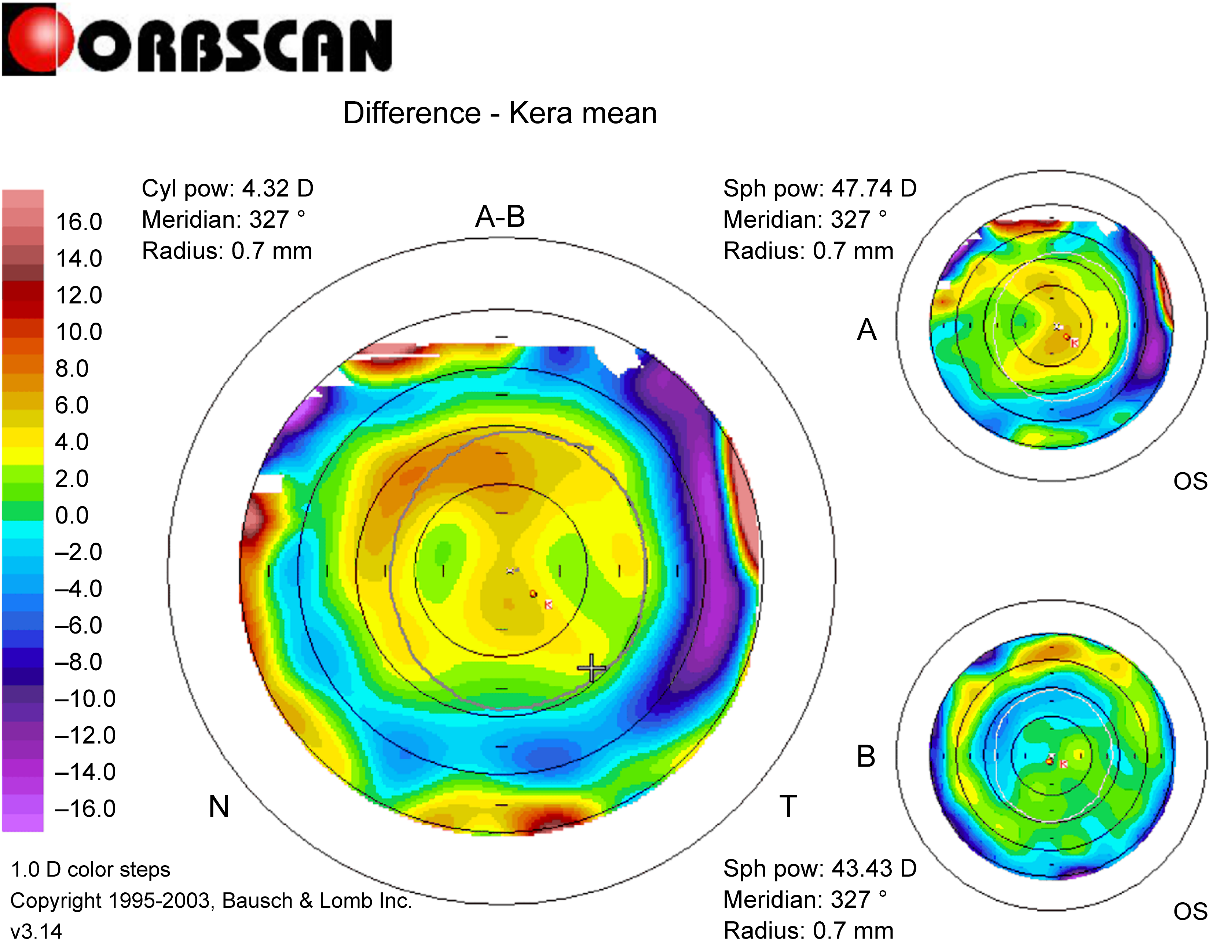
**

**Figure S1.** Differential pachymetric map comparing pre- and post-L-LAK in Case 1.
